# Venous and Arterial Thromboembolism in Lung Cancer Patients: A Retrospective Analysis

**DOI:** 10.3390/jcm13133773

**Published:** 2024-06-27

**Authors:** Olga Morath, Julia Hoffmann, Kristina Schilling, Andreas Hochhaus, Tobias Rachow, Susanne M. Lang

**Affiliations:** 1 Klinik für Innere Medizin II, Hämatologie und Internistische Onkologie, Universitätsklinikum Jena, Am Klinikum 1, 07747 Jena, Germany; kristina.schilling@med.uni-jena.de (K.S.); andreas.hochhaus@med.uni-jena.de (A.H.); 2 Klinik für Innere Medizin V, Pneumologie, Universitätsklinikum Jena, Am Klinikum 1, 07747 Jena, Germany; julia.hoffmann2@med.uni-jena.de (J.H.); susanne.lang@med.uni-jena.de (S.M.L.); 3 Internistisch-Onkologische Gemeinschaftspraxis, Wiesestrasse 22, 07548 Gera, Germany; t.rachow@iogp.de

**Keywords:** venous thromboembolism, arterial thromboembolism, lung cancer, immune checkpoint inhibitors, platinum-based therapy

## Abstract

**Background**: Patients with lung cancer face an increased incidence of venous (VTE) and arterial (ATE) thromboembolism. Risk factors for thrombosis remain unclear, particularly the impact of the use of immune checkpoint inhibitors (ICIs). We sought to compare the incidence of VTE and ATE in lung cancer patients receiving platinum-based therapy versus those receiving ICIs alone or in combination with chemotherapy and to validate the Khorana risk score for predicting VTE in the era of ICIs. **Methods**: A retrospective single-institution data analysis of 173 patients diagnosed with locally advanced or metastatic lung cancer at the Jena University hospital between 2015 and 2021. **Results**: The study revealed a high incidence of VTE (17.9%) and ATE (5.8%). The VTE risk was higher in patients diagnosed with adenocarcinoma (OR 0.29, 95% CI 0.09–0.93) than in patients with other histological types. A prior venous event was associated with an increased risk of recurrent VTE (OR 4.46, 95% CI 1.20–16.63). The incidence of thrombosis under first-line platinum-based chemotherapy did not differ from the incidence under ICIs (*p* = 0.19). There were no differences in the subgroup of patients who received ICIs alone or combined immunochemotherapy (*p* = 0.43). The Khorana score failed to predict the risk of VTE correctly. **Conclusions**: We did not find evidence supporting the theory that ICI therapy (alone or combined) increases the risk of thrombotic events. Adenocarcinoma and a prior history of VTE were strongly associated with an increased risk of VTE. Other scores for thrombotic risk assessment in lung cancer patients should be tested in prospective studies.

## 1. Introduction

Patients diagnosed with advanced lung cancer face a high risk of venous (VTE) and arterial (ATE) thromboembolism, as evidenced by various studies [1,2,3,4]. These cancer-associated thrombotic events are not only associated with increased mortality rates but also lead to expanded healthcare costs, a heightened risk of bleeding, and potential delays in cancer treatment. Thrombotic complications rank as the second most frequent cause of death among tumor patients, with cancer progression standing as the primary cause [5,6,7]. In the landscape of immuno-oncology research, it has become essential to understand whether ICI therapy contributes to thrombotic risk, and which risk factors remain the most important in the new era of modern systemic therapy. A better understanding of the pathophysiology of thrombosis is essential for developing prophylactic and treatment strategies and improving patient outcomes in cancer care.

Based on recent studies, we summarized in Table 1 the potential triggers for VTE and ATE in advanced lung tumors [1,7,8,9,10,11,12,13,14,15,16,17]. However, determining which of these factors exerts the most powerful thrombogenic effect remains a subject of ongoing research.

The VTE incidence in patients with lung tumors undergoing platinum-based systemic therapy ranges from 6% to 13% [18] and is associated with low overall survival [8,10,19,20]. Platinum-based chemotherapy regimens are known to cause dysfunction in endothelial cells, which results in proinflammatory changes and a high expression of cell adhesion molecules, thereby elevating the risk of VTE [14,21,22,23]. Since 2015, the development of immuno-oncology has shifted cancer treatment [24], initially improving survival outcomes in patients with non-small cell lung cancer (NSCLC) and subsequently in small cell carcinoma (SCLC) as well. In light of the close link between the activation of the immune system, inflammation, platelet adhesion, and thrombosis, immune checkpoint inhibitors (ICIs) could potentially promote thrombotic events [24,25,26,27]. However, prior studies have shown contradictory results regarding the association between thrombosis incidence and ICI treatment [8,19,25,28,29,30,31,32,33,34,35,36,37,38]. Most of this research involved patients with different malignancies or stages, medical history, baseline medication, particularly antithrombotic drugs, prior anticancer treatment, and no control group.

A significant bias in studies on cancer treatment and thrombotic risk is the inclusion or exclusion of patients on anticoagulants or antiplatelet drugs before starting systemic therapy [8,28]. To correctly assess the direct impact of comprehensive therapy on thrombotic risk, patients on antithrombotic treatment at the baseline should ideally be excluded. However, this is impractical and not possible in real-world scenarios, as many patients develop thrombosis before or at cancer diagnosis. Thrombosis often serves as an early indicator of tumor activity, complicating the exclusion of these patients from studies [39]. Thus, real-world research frequently includes patients with preexisting thrombotic conditions, unfortunately influencing study outcomes on the thrombotic risks of systemic treatments. On the other hand, it is challenging to ensure that a patient receives the appropriate oral anticoagulant dose during systemic therapy. Inadequate dose of anticoagulant and as a result increased risk of recurrent VTE or ATE might be caused by postponing or dose reductions in the context of advanced cancer and anticancer therapy side effects like vomiting, poor nutrition, and reduced performance index as well as cognitive impairment.

Next, the lack of a control group in recent analyses is a significant issue. It can be highly difficult to compare patients with lung cancer who have received distinct treatment regimens. Most patients receiving different regimens have unique characteristics that influence the decision about therapy, such as molecular characteristics of the tumor, Eastern Cooperative Oncology Group (ECOG) performance status, or comorbidities. As a result, we chose to compare primarily the same patients who were receiving chemotherapy and ICIs as a next-line treatment. It was clear to us that patients in this case may be at a higher thrombotic risk as their disease progresses.

Additionally, the thrombotic risk differs between outpatients and hospitalized patients undergoing systemic therapy. The majority of patients undergoing chemotherapy in hospitals require prophylactic doses of low-molecular-weight heparin (LMWH) for the duration of the hospitalization [40]. Consequently, we analyzed outpatients to reduce bias between these patient groups. Outpatients with lung cancer are not routinely considering thrombosis prevention medication. To answer the question of whether outpatients with lung cancer, particularly those in advanced stages, require thrombosis prophylaxis in the new era of immuno-oncology, a reliable thrombotic risk score must be established.

The Khorana risk score was validated in an observational cohort of cancer outpatients starting chemotherapy [41]. It has emerged as a critical component in determining which patient should receive VTE thromboprophylaxis. However, there is limited evidence regarding VTE risk assessment and the effectiveness of thromboprophylaxis in tumor patients undergoing comprehensive systemic treatment, particularly ICI therapy. Additionally, a recent study has shown that the Khorana score has limited power in distinguishing between lung cancer patients at intermediate or high risk of VTE [42].

Given the conflicting findings of recent studies on the incidence of thrombosis under modern systemic treatment, this real-world study aimed to compare the incidence of VTE and ATE in lung cancer patients receiving platinum-based therapy versus those receiving ICIs alone or in combination with chemotherapy. The risk factors for VTE and ATE have been investigated separately. Furthermore, this study sought to validate the Khorana risk score’s ability to predict VTE in patients receiving immune checkpoint inhibitor therapy.

## 2. Materials and Methods

A retrospective data analysis of patients diagnosed with locally advanced or metastatic lung cancer at the Jena University hospital between 2015 and 2021 was conducted. Patients were included if they received at least 1 cycle of cancer therapy: anti-PD-1, anti-PD-L1, anti-CTLA4-antibodies, a combination of these agents, or a combination of chemotherapy with any of these drugs during first-line or further-line therapy. Data (demographic characteristics, performance status, smoking, thromboembolic events, lung function, comorbidities, central line, type, and line of therapy received) were collected from routine medical records. Imaging methods confirmed the diagnosis of VTE, which included symptomatic or incidental deep vein thrombosis, pulmonary embolism, splanchnic, and catheter-related thrombosis. ATE was defined as ischemic stroke, acute coronary syndrome, or acute peripheral artery occlusion. Thrombotic events were considered therapy-related when at least one cycle of systemic therapy had been applied. The overwhelming majority of patients (>92%) received systemic therapy in the outpatient clinic department. To represent a real-world scenario, patients with a history of thrombotic events or those who received continuous anticoagulation and antiplatelet therapy were not excluded. Routine laboratory data (complete blood count, global coagulation assays, lactate dehydrogenase, albumin, and cholesterol levels) were collected at the beginning of systemic therapy. From these data, the Khorana risk score was calculated using five clinical and pre-chemotherapy laboratory parameters: the primary tumor site (+1 for lung cancer), a platelet count of 350 × 109/L or more, a hemoglobin concentration of 100 g/L or lower, or the use of erythropoiesis-stimulating agents, a leukocyte count of 11 × 109/L or higher, and a body mass index of 35 kg/m^2^ or higher. Patients with a sum score of 0 are classified as having a low risk of VTE, those with 1 or 2 as having an intermediate risk, and those with 3 or more as having a high risk.

Variables were reported as median with interquartile range (IQR). Medians of the two groups were compared using the Wilcoxon–Mann–Whitney test. Categorical variables were reported as counts and proportions and compared using chi-square or Fisher’s exact test. Univariable and multivariable logistic regression analyses were used to identify independent predictors of VTE and ATE. Median follow-up and overall survival (OS) were estimated using the Kaplan–Meier method. Cox regression was used for univariable and multivariable analysis of survival predictors. All reported *p*-values are two-sided, and *p* < 0.05 was considered statistically significant. SPSS version 28 was used for statistical calculations.

## 3. Results

In the period between 2015 and 2021, we identified 173 patients who were treated with any immunotherapy. In total, 72 out of 173 (41.6%) patients were still alive at the moment of the data cut-off. Median follow-up and median OS were 27 (95% CI 20.6–33.4) months and 17 (95% CI 13.9–20.2) months, respectively. The most common histological type of lung cancer was adenocarcinoma (*n* = 98, 56.6%), followed by squamous cell carcinoma (*n* = 47, 27.2%) and small cell carcinoma (*n* = 21, 12.1%). Around 62 (43.7%) patients received cisplatin and 80 (56.3%) patients received carboplatin. The most frequently used ICI was pembrolizumab (*n* = 89, 51.4%) followed by nivolumab (*n* = 36, 20.8%) and atezolizumab (*n* = 36, 20.8%), durvalumab (*n* = 11, 6.4%), and ipilimumab in combination with nivolumab (*n* = 1, 0.6%). A total of 88 (50.9%) patients received combined immunochemotherapy. Table 2 includes information on cancer diagnosis, patient characteristics, and treatments. During follow-up, 31 (17.9%) patients developed VTE, and 10 (5.8%) patients developed ATE. Localizations of thromboembolic events are shown in Table 3. One patient experienced a fatal pulmonary embolism.

### 3.1. Incidence and Risk Factors of VTE and ATE

The incidences of thrombosis stratified by histological subtype are summarized in Table 4.

Patients with VTE during follow-up (*n* = 31) and those who had no thrombotic events (*n* = 142) were compared. The risk of VTE exhibited a notable disparity between patients diagnosed with adenocarcinoma compared to those with squamous cell carcinoma (OR 0.29, 95% CI 0.09–0.93, *p* = 0.03). The level of programmed death ligand 1 (PD-L1) expression on tumor cells did not demonstrate a correlation with thrombotic risk (*p* = 0.48). Prior VTE was associated with recurrent venous events (OR 4.46, 95% CI 1.20–16.63, *p* = 0.03). No association was identified between age, sex, Eastern Cooperative Oncology Group (ECOG) performance status, or laboratory parameters and the incidence of VTE. Table 5 and Table 6 reveal the results of the risk factor exploration for VTE. There was a statistically significant association between the incidence of ATE and smoking status (*p* = 0.005).

### 3.2. VTE and ATE during Platinum-Based Chemotherapy vs. ICI Treatment

The incidence of thrombotic events under first-line platinum-based chemotherapy (group 1) was compared to the incidence under ICIs alone or combined ICIs and chemotherapy (group 2). No difference between the two groups was found (*p* = 0.19). There were no differences in the number of thrombotic events in the subgroup of patients who received ICIs alone (*n* = 85) or combined immunochemotherapy (*n* = 88) (*p* = 0.43). We observed no significant difference in thrombosis incidence between patients receiving cisplatin and those receiving carboplatin (*p* = 0.33).

### 3.3. Subgroup Analyses—Incidence of Thrombotic Events in Patients with or without Antithrombotic Therapy at Baseline

Prior VTE or atrial fibrillation were indications for continuous anticoagulation at baseline. Four patients received vitamin K antagonists, fifteen patients—direct oral anticoagulants (DOACs), seven patients—prophylactic doses of low-molecular-weight heparin (LMWH), seven patients—intermediate-dose LMWH, and five patients received therapeutic-dose LMWH. Prior ATE was an indication for antiplatelet therapy at baseline in 31 of 33 cases. The indication was not reported in the medical charts of two patients. Low-dose aspirin was administered to thirty-one patients and the P2Y12 inhibitor clopidogrel to two patients. No relevant hemorrhage was documented during antithrombotic therapy.

Two groups of patients were compared: those who received anticoagulation prior to initiating ICI treatment (*n* = 38) versus those who did not (*n* = 135). No association with VTE risk was observed for patients undergoing continuous anticoagulation (*p* = 0.68).

### 3.4. Khorana Risk Score

In total, 94 (54%) patients had one point on the Khorana risk score, 50 (29%) had two, indicating intermediate risk, and 29 (17%) had three or more points, indicating high risk. The Khorana score failed to correctly predict the risk of VTE in patients who received classic platinum-based systemic treatment (*p* = 0.20) or ICIs (*p* = 0.54).

### 3.5. Survival of Patients with Thrombosis Versus No Thrombosis

**Figure 1:**
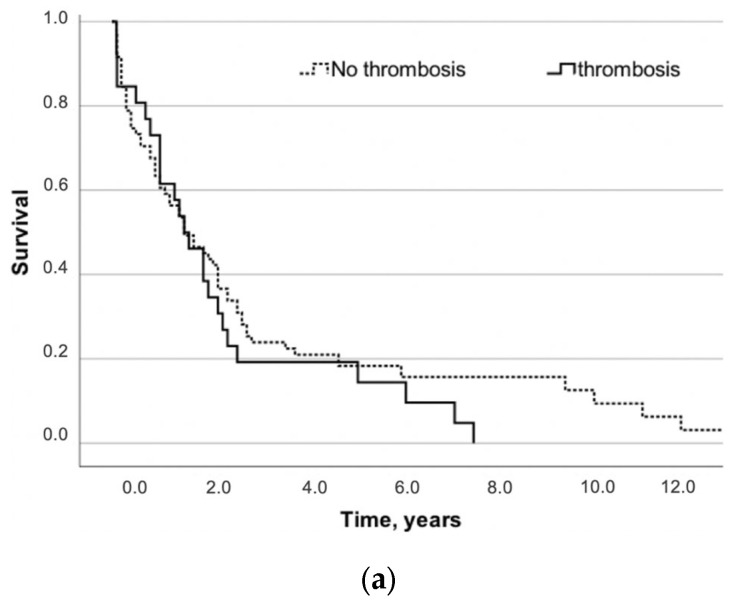

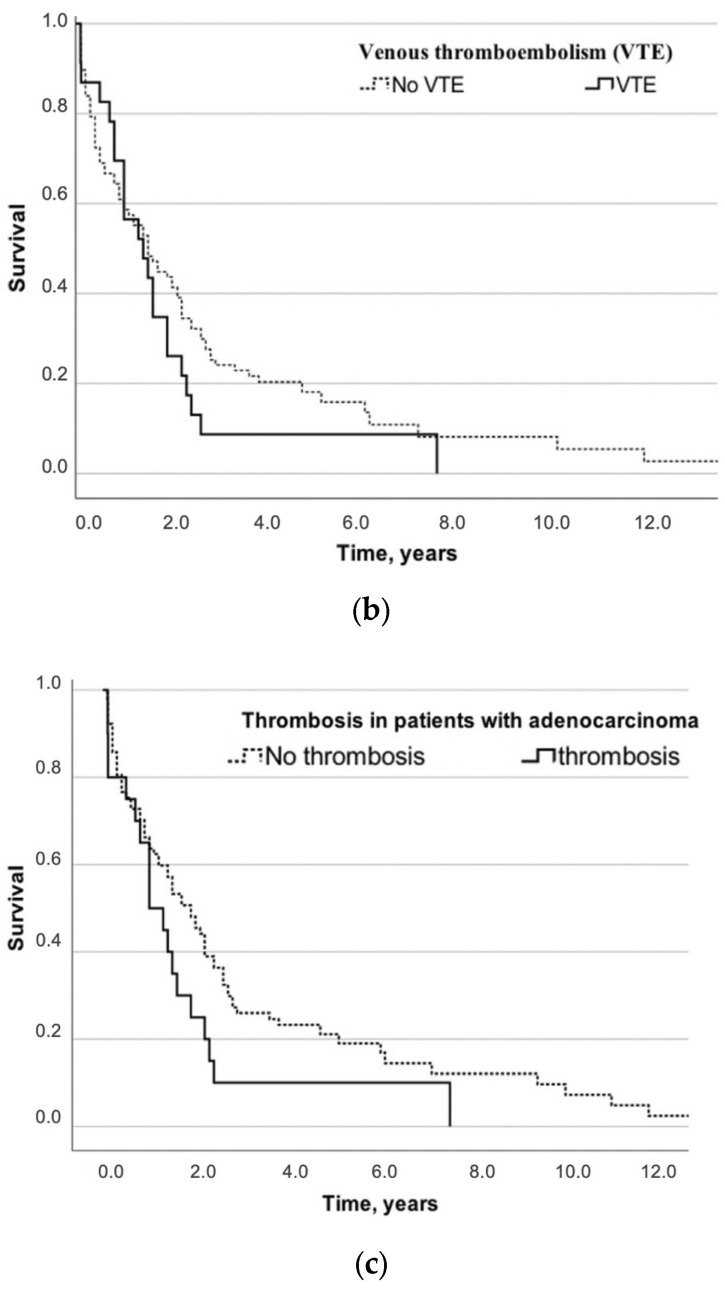
(**a**) Survival of NSCLC patients with thrombosis vs. no thrombosis. (**b**) Survival of NSCLC patients with VTE vs. no VTE. (**c**) Survival of patients with adenocarcinoma with thrombosis vs. no thrombosis.

Patients with thrombosis in the follow-up had a similar OS to patients without thrombosis (*p* = 0.32) (Figure 1a–c). In a multivariable analysis, female sex (HR for male vs. female 0.62, 95% CI 0.39–0.99, *p* = 0.04) and blood transfusion (HR 2.13, 95% CI: 1.21–3.72, *p* = 0.008) were found to be negative prognostic factors.

## 4. Discussion

This real-world study examined the incidence and risk factors associated with venous and arterial thromboembolism in lung cancer patients undergoing treatment with platinum-based therapy or receiving ICI monotherapy or in combination with traditional platinum-based regimens. The study revealed a high incidence of VTE (17.9%) and ATE (5.8%) in patients with advanced lung cancer, which was similar to or higher than in previously published data [13,17,42,43].

Compared to patients with other histological types, patients with adenocarcinoma had a significantly higher risk of VTE. It has been observed that malignant mucins stimulate platelets and produce microthrombi in mice; this is probably due to their interaction with platelet P-selectin and leukocyte L-selectin. The higher incidence of VTE in patients with lung adenocarcinoma may be explained by this interaction between selectins and circulating carcinoma mucins [17,44]. Prior thrombosis is an established risk factor for recurrent thrombosis in cancer patients [8,32]. In this study, this risk factor was confirmed for patients with advanced lung tumors undergoing classic chemotherapy or immunotherapy.

Traditionally, conventional cytotoxic chemotherapy, especially cisplatin, is known to elevate the risk of VTE [14,23]. Carboplatin-based regimens are commonly utilized as an alternative to cisplatin combination chemotherapy for patients who are deemed unfit. However, in the recent study, the occurrence of VTE related to platinum therapy remained high, and we investigated no statistical difference in VTE risk between the cisplatin and carboplatin groups, consistent with recent findings [11]. The observed thrombotic event incidence of 8.1% during ICI treatment in this study aligns with recent research findings, which have reported incidences ranging from 4.5% [45] to 14.8% [19]. In contrast to previous research [8,28], our findings do not support the hypothesis that patients receiving ICI treatment have a higher risk of thrombotic events than patients receiving conventional chemotherapy. It is crucial to note that our patients mostly received ICI treatment not as a first-line (1L) option (mostly 2L, up to 7L), but rather after experiencing disease progression under conventional chemotherapy. One possible explanation for the lower event rate observed with ICIs is the continued use of anticoagulants before initiating immunotherapy. However, we found no statistical correlation between patients who received anticoagulants prior to ICI treatment and those who did not. Furthermore, this suggests that cancer itself is the major trigger of thrombosis, regardless of the type of systemic therapy administered.

The recommendation for medicinal thrombosis prophylaxis in patients with lung cancer remains an individual decision with special consideration of the bleeding risk. In our study, we examined the efficacy of the Khorana risk score to determine VTE, which has traditionally been used for evaluating VTE risk in the ambulatory health care system prior to systemic therapy beginning [41]. Similar to previous findings [8,9,31,46], the Khorana score did not predict the risk of VTE correctly in patients receiving ICIs. We propose that other scores (ONCOTEV or COMPASS-CAT) might be a more convenient tool for thrombosis risk stratification in patients with lung cancer receiving modern treatment and should be tested in prospective studies for a better prophylactic strategy [47,48]. Based on the findings in this and recent studies, it is clear that new thrombotic risk assessment scores for patients with lung tumors should include additional risk factors such as histology and prior VTE.

The risk of ATE was associated with a well-known cardiovascular disease risk factor: smoking status. According to the most recent lung cancer epidemiologic study, quitting smoking improves overall survival, particularly among women [49]. Based on these findings, lung cancer patients should be advised to modify their lifestyle.

Conflicting data regarding decreased survival subsequent to VTE in cancer patients receiving systemic therapy have been reported in previous research [8,10,19]. In the current study, no significant correlation between the occurrence of thrombosis and OS was observed. Reasons for treatment termination were disease progression, respiratory failure, immune-related adverse events, or infections. In a multivariate analysis, female sex and blood transfusion were associated with poorer survival. Anemia has been linked to poorer survival rates, disease progression, chemotherapy delays, lower anticancer treatment responses, and decreased blood oxygen transport capacity [50].

## 5. Conclusions

In summary, our patient cohort with advanced lung cancer was at high risk of developing VTE and ATE. The incidence of thrombotic events under platinum-based therapy, ICIs alone, or combined chemoimmunotherapy did not differ significantly. We found that patients with adenocarcinoma and a prior history of VTE had a particularly high risk for VTE. The Khorana score did not predict the risk of VTE in this cohort correctly. Other scores should be tested in prospective studies to determine a better prophylactic strategy, with histology and prior VTE as additional risk factors. The occurrence of thrombotic events had no impact on overall survival.

The study has significant limitations, including its retrospective single-center design and confounding bias. Patients who had received antithrombotic drugs at the baseline were not excluded. Further prospective studies are required to identify risk factors and biomarkers to more completely comprehend and foresee the risk of VTE and ATE in patients receiving contemporary lung cancer treatment.

## Figures and Tables

**Table 1:** Risk factors for thromboembolic events in lung cancer patients.

Patient-Related Factors	Tumor-Related Factors	Therapy-Related Factors
Age	Tumor stage Histologic subtype Biomarkers (e.g., PD-L1, oncogenic driver mutation, cancer cells expressing high levels of tissue factor and systemic tissue factor)	Platinum-based therapy
Sex		Antiangiogenic therapy (e.g., VEGF inhibitors)
Smoking history		Immune checkpoint inhibitor therapy
Performance status		Central venous line
Body mass index (BMI)		Surgical procedure
Comorbidities (e.g., COPD, diabetes or coronary heart disease, autoimmune and renal diseases, infection)		Hospitalization
History of thromboembolic events		Transfusion

COPD = chronic obstructive pulmonary disease, PD-L1 = programmed cell death ligand−1, VEGF = vascular endothelial growth factor.

**Table 2:** Patient baseline characteristics (*n* = 173).

Characteristics	*n* Patients/Events (%)
Demographics and clinical characteristics	
Age, years (median, range)	65 [57–72]
Sex, male/female	111 (64.2%)/62 (35.8%)
ECOG ≥ 2	21 (12.2%)
Smoking	126 (72.8%)
pack years (median, range)	35.0 [20.0–46.3]
Diabetes mellitus	42 (24.3%)
Coronary artery disease	17 (9.8%)
Arterial hypertension	107 (61.8%)
Forced Expiratory Volume in 1 Second (FEV1), l (median, range)	2.43 [1.9–3.2]
Peripherally inserted central venous catheter (PICC)	33 (19%)
Port	102 (59%)
Prior venous thromboembolism (>2 years before cancer diagnosis)	12 (6.9%)
Venous thromboembolism under chemotherapy	19 (10.6%)
Venous thromboembolism under checkpoint inhibitors	12 (6.9%)
Prior arterial thromboembolism (>2 years before cancer diagnosis)	31 (17.3%)
Arterial thromboembolism under chemotherapy	8 (4.6%)
Arterial thromboembolism under checkpoint inhibitors	2 (1.2%)
Continuous anticoagulation at baseline	30 (17.3%)
Continuous antiplatelet therapy at baseline	25 (14.5%)
Combined continuous anticoagulation and antiplatelet therapy	8 (4.6%)
Khorana risk score	
1–2	144 (83%)
3–4	29 (17%)
Tumor characteristics	
Histology	
Adenocarcinoma	98 (56.6%)
Squamous cell carcinoma	47 (27.2%)
Small cell carcinoma	21 (12.1%)
Undifferentiated and others	7 (4.0%)
PD-L1 expression	
<1%	37 (21.4%)
1–49%	45 (26.0%)
≥50%	48 (27.7%)
Therapy	
Initial surgical therapy	40 (23.1%)
Initial radiation therapy	62 (35.8%)
Cisplatin	62 (35.8%)
Carboplatin	80 (46.2%)
Concomitant therapy during checkpoint inhibitors therapy (*n*)	88
Combination with carboplatin	66 (75.0%)
Combination with other chemotherapy or targeted therapy	22 (25.0%)
Pembrolizumab	89 (51.4%)
Nivolumab	36 (20.8%)
Atezolizumab	36 (20.8%)
Durvalumab	11 (6.4%)
Ipilimumab and nivolumab	1 (0.6%)
Line of therapy where ICI was applied	2, range 1–7

PD-L1 = programmed cell death ligand−1, ICI = immune checkpoint inhibitor, ECOG = Eastern Cooperative Oncology Group performance status.

**Table 3:** Clinical manifestation of VTE and ATE.

	*n* (%) Patients
Deep vein thrombosis	11/173 (6.4%)
Deep vein thrombosis and pulmonary embolism	6/173 (3.5%)
Catheter-related thrombosis	6/173 (3.5%)
Pulmonary embolism	5/173 (2.9%)
Splanchnic thrombosis	2/173 (1.2%)
Sinus vein thrombosis	1/173 (0.6%)
In total, venous thrombotic events	31/173 (18.1%)
Acute coronary syndrome	4/173 (2.3%)
Ischemic stroke	3/173 (1.7%)
Acute vascular occlusion	3/173 (1.7%)
In total, arterial thrombotic events	10/173 (5.7%)

**Table 4:** Rate of thrombotic events according to histological subtype.

Thrombotic Event	Small Cell Carcinoma, *n*/N (%)	Adenocarcinoma, *n*/N (%)	Squamous Cell Carcinoma, *n*/N (%)
VTE under chemotherapy	0	17/98 (17.4%) *	2/47 (4.3%)
VTE under ICIs	1/21 (4.8%)	9/98 (9.2%)	2/47 (4.3%)
ATE under chemotherapy	1/21 (4.8%)	3/98 (3.1%)	4/47 (8.5%)
ATE under ICIs	1/21 (4.8%)	1/98 (1.02%)	0

* *p* = 0.03; VTE = venous thromboembolism, ATE = arterial thromboembolism, ICI = immune checkpoint inhibitor.

**Table 5:** Investigation of VTE risk factors in univariable analysis.

Risk Factor	*p* Value
Age	0.29
Sex	0.65
Eastern Cooperative Oncology Group (ECOG) performance status	0.96
Smoking	0.78
Diabetes mellitus	0.60
Coronary artery disease	0.48
Arterial hypertension	0.65
Forced Expiratory Volume in 1 Second (FEV1)	0.27
Prior venous thromboembolism (>2 years before cancer diagnosis)	<0.001
Khorana risk score	0.20
Histology (adenocarcinoma versus squamous cell carcinoma)	<0.001
PD-L1 expression	0.48
Cisplatin versus carboplatin	0.33
Chemotherapy versus ICI therapy (alone or combined)	0.19
ICIs alone versus combined immunochemotherapy	0.43

PD-L1 = programmed cell death ligand−1, ICI = immune checkpoint inhibitors.

**Table 6:** Investigation of VTE risk factors in multivariable analysis.

Risk Factor	Multivariable Analysis, OR, 95% CI, *p* Value
Prior venous thromboembolism (>2 years before cancer diagnosis)	OR 4.46, 95% CI 1.20–16.63, *p* = 0.03
Histology (adenocarcinoma versus squamous cell carcinoma)	OR 0.29, 95% CI 0.09–0.93, *p* = 0.03

## Data Availability

The data presented in this study are available upon request from the first author.
